# Assessment of Nurses’ Knowledge Regarding Pressure Injury: A National Multicenter Cross-Sectional Study

**DOI:** 10.3390/healthcare14131948

**Published:** 2026-07-01

**Authors:** Ana Žepina Puzić, Bojana Filej

**Affiliations:** 1 Faculty of Health Sciences, University of Novo Mesto, 8000 Novo Mesto, Slovenia; 2 Department of Health Studies, Šibenik University of Applied Sciences, 22000 Šibenik, Croatia

**Keywords:** nursing education, nursing knowledge, Pieper–Zulkowski Pressure Ulcer Knowledge Test, pressure injury, education, pressure ulcer prevention

## Abstract

**Highlights:**

**What are the main findings?**

- Nurses’ knowledge regarding the prevention and management of pressure injuries differed according to their educational level, work environment, and engagement in educational activities, although the observed effect sizes were generally small. No statistically significant differences were found according to the frequency of working with patients with pressure injuries.

**What are the implications of the main findings?**

- The findings provide a national overview of nurses’ knowledge of pressure injuries and may serve as a basis for planning future educational activities. Additional longitudinal and intervention studies are needed to evaluate the effectiveness of different educational approaches and their relationships with clinical practice.

**Abstract:**

**Background/Objectives:** Improving the prevention and management of pressure injuries requires adequate nursing knowledge. In this context, understanding the current knowledge levels of nurses is important for informing educational and organizational strategies. This study aimed to assess the pressure injury knowledge of nurses at the national level and examine differences according to their educational, professional, and informational characteristics. **Methods:** A national cross-sectional quantitative study was conducted in public secondary-level hospitals in the Republic of Croatia. A total of 1139 participants from 19 hospitals across all four geographical regions of Croatia participated. The PZ-PUNKT instrument was used, and an analysis was conducted using descriptive and bivariate inferential statistics. **Results**: Participants with bachelor’s and master’s degrees achieved higher PZ-PUKT scores than those with secondary education (*p* = 0.026 to < 0.001), although the effect sizes were very small (ε^2^ = 0.008–0.017). Significant differences were observed across clinical departments (*p* < 0.001; ε^2^ = 0.03–0.04), whereas no statistically significant differences were found according to the frequency of working with patients with pressure injuries (*p* > 0.05). Participants who reported recently attending educational activities, consulting the professional literature, or searching for information achieved higher knowledge scores across all domains (*p* < 0.001); however, the effect sizes remained small (ε^2^ = 0.034–0.060; rpb = 0.133–0.214). **Conclusions:** Although the observed effect sizes were generally small, higher knowledge scores were observed among nurses who reported recent engagement with educational activities, the professional literature, and information-seeking behaviors. No significant differences were identified according to the frequency of working with patients with pressure injuries. These findings provide a national overview of pressure injury knowledge among Croatian nurses and may inform future educational initiatives and research.

## 1. Introduction

Pressure injury (PI) represents a complex clinical phenomenon that is considered an indicator of the quality of healthcare [1]. Its incidence and prevalence at various levels of healthcare, ranging from acute hospital facilities to long-term care institutions, underscore the need for systematic and evidence-based approaches to relevant preventive and curative measures. In this context, PI is viewed as the result of a dynamic interaction of biological, organizational, and professional factors with the capacities and characteristics of the affected person [2].

Treatment represents a significant financial burden for the healthcare system, especially in the advanced stages of disease, where costs increase significantly and often exceed previous estimates [3]. Despite the large amount of available scientific evidence, the incidence and prevalence of PI remain significant, indicating a gap between the available evidence and its implementation in clinical practice. Preventive interventions such as regular patient repositioning, the use of pressure relief surfaces, and ensuring adequate nutritional support have been shown to reduce the incidence of PI [4]. However, their effectiveness in clinical practice largely depends on consistent application, interdisciplinary collaboration, and organizational support. In this sense, work protocols and institutional guidelines play a key role in standardizing procedures and reducing variability in care [5].

An important factor in the successful implementation of preventive and therapeutic measures is the level of knowledge of healthcare professionals, especially nurses, who play a key role in the daily care of patients. In the Republic of Croatia, nurses can obtain professional qualifications through a five-year secondary nursing education program following primary education or through university undergraduate and graduate nursing studies, aligned with the Bologna process and European directives [6]. Upon completion of secondary nursing education, nurses are eligible for direct entry into the Croatian Nursing Council registry in accordance with defined competencies. Although this level of education does not include a higher education degree, it comprises theoretical instruction and clinical practice in accordance with European standards for nursing education. Admission to undergraduate nursing studies requires the completion of four-year secondary education and passing the national state examination, while admission to graduate nursing studies requires the completion of an undergraduate nursing degree and acquisition of 180 ECTS credits [7].

The level of knowledge of nurses is shaped by interactions between their formal education, clinical experience, organizational environment, and ability to access, evaluate, and apply expert information. Formal education provides a theoretical basis for understanding the etiology, prevention, and treatment of PI, while the clinical environment enables the application and upgrading of knowledge through everyday practice [8]. At the same time, the capability to actively search for, critically appraise, and apply scientific evidence is a key component of modern evidence-based nursing practice, contributing to continuous professional development and quality clinical decision-making.

The impact of education on the level of knowledge represents one of the fundamental dimensions of professional development in healthcare [9]. Knowledge is not viewed solely as the accumulation of information, but as the integration of theoretical knowledge, clinical reasoning, and the ability to apply guidelines in complex care situations [10].

However, knowledge is only one component of professional competence. Contemporary nursing competency models define competence as the integration of knowledge, practical skills, professional judgment, communication skills, and professional behaviors in real-world clinical situations. However, existing knowledge assessment instruments—including the PZ-PUKT—primarily assess the cognitive component of competence and do not allow for direct assessment of the quality of clinical performance or patient care outcomes. The results of such instruments should therefore be viewed as indicators of the level of knowledge, rather than as a direct measure of overall professional competence [11].

Empirical evidence indicates that structured educational interventions, including formal education and continuing professional development, lead to significant increases in the level of knowledge of nurses. The most pronounced effects are recorded in programs that combine theoretical background, clinical case analysis, and competency evaluation [12]. However, the level and sustainability of the acquired knowledge depend on the type of education, teaching methods, and the possibilities for practical application of the content. Increasing the level of knowledge in itself does not necessarily result in changes in clinical practice; the application of knowledge also depends on organizational factors, availability of resources, the culture of the work environment, leadership support, and opportunities for continuous professional development. The lack of coordinated education and limited implementation of guidelines often leads to inconsistent practice and poorer clinical outcomes [13]. In the modern health system, decision-making for the prevention and treatment of PI is based on the principles of evidence-based practice, with health information technologies playing an increasing role in supporting clinical decision-making [14]. Professional guidelines emphasize the importance of continuous monitoring of the knowledge and competencies of healthcare professionals and the need for continuing education of nurses [15].

Despite the available scientific knowledge, a comprehensive understanding of the impact of nurses’ knowledge levels on the consistent application of preventive and therapeutic guidelines in clinical practice is lacking. A particular problem is the lack of systematic national data on the level of knowledge of nurses regarding the prevention and treatment of PI in the Republic of Croatia. Although international research has indicated significant differences in the level of knowledge between health systems, these results cannot be directly transferred to the Croatian context due to differences in educational models, healthcare organization, regulatory frameworks, and the professional competencies of nurses. In this regard, national data are particularly useful as they allow for identification of the specific educational needs of Croatian nurses, assessment of the effectiveness of existing educational models, and planning of targeted interventions to improve the quality of care. Such a research gap thus limits the possibility of developing targeted educational interventions and improving the quality of care.

Therefore, the aim of this study is to assess the level of knowledge regarding PI among nurses actively participating in clinical practice in the Republic of Croatia. This study assesses the level of knowledge of nurses with respect to their level of education (secondary school, undergraduate, or graduate), in accordance with the particular educational structure for nurses in the Croatian healthcare system [16]. Furthermore, specific gaps and concerns related to this topic are identified, and the relationships between the level of knowledge and relevant professional and educational factors are analyzed. The obtained results are expected to contribute to a better understanding of the educational and professional factors associated with nurses’ knowledge of PI, serving as a basis for planning future educational interventions and strategies to improve the quality of care in the Croatian healthcare system.

## 2. Materials and Methods

### 2.1. Study Design and Setting

This study was conducted as a national cross-sectional study in the Republic of Croatia, Europe. It included nurses from 19 of the country’s 22 public secondary-level hospitals, providing broad national coverage. This cross-sectional study assessed nurses’ knowledge regarding PI, with particular emphasis on differences according to their educational level and professional education. The study analyzed knowledge related to the prevention, classification, and description of PI, as well as differences in knowledge results with respect to relevant educational and professional characteristics of the respondents. The results are reported in accordance with the STROBE guidelines for observational studies.

### 2.2. Research Instrument and Reliability

The Croatian version of the Pieper–Zulkowski Pressure Ulcer Knowledge Test, Version 2 (PZ-PUKT) was used in this study to assess the respondents’ knowledge of PI. This instrument was previously translated, culturally adapted, and psychometrically validated according to established cross-cultural adaptation procedures. The validation study demonstrated excellent content validity (S-CVI = 0.981), acceptable internal consistency (KR-20 = 0.79), high split-half reliability (Guttman’s Lambda 6 = 0.89), and excellent test–retest reliability (ICC = 0.91), supporting its use among Croatian healthcare professionals [17]. Although the instrument provides an objective assessment of PI-related knowledge, it should not be considered a direct measure of overall clinical competence, which additionally depends on practical skills, clinical reasoning, and contextual clinical factors.

The questionnaire used in this study consists of two parts. The first part includes sociodemographic questions and items related to sources and methods of knowledge acquisition. The second part contains 72 items from the PZ-PUKT test, organized into three subscales—prevention (31 items), wound description (20 items), and staging (21 items)—with an additional overall score calculated. Participants respond to each item with “True”, “False”, or “Don’t know”. The total score on the questionnaire ranges from 0 to 72 points. According to the interpretation criteria proposed for the original PZ-PUKT instrument, scores below 70% indicate an unsatisfactory level of knowledge, scores between 70% and 79.9% reflect a satisfactory level, scores from 80% to 89.9% indicate good knowledge, and scores of 90% or higher represent a very good level of knowledge regarding PI [18].

### 2.3. Data Collection

Data collection was carried out using a paper-based version of the questionnaire following approval by the relevant ethics committees. The process took place between June and December of 2025. All participants were informed about the aims and purpose of the study and provided written informed consent prior to participation. Completion of the questionnaire was anonymous and voluntary, with an average completion time of approximately 25–30 min. Only fully completed questionnaires were included in the final analysis, ensuring data completeness and reliability of the calculated total and subscale scores. After obtaining approval from the relevant ethics committees, the research team—in collaboration with the assistant directors of nursing and the head nurses of the institutions—organized the implementation of the study and the distribution of the questionnaires in the clinical departments of the participating hospitals. The nurses were informed about the voluntary nature of the study, and no financial or other incentives for participation were used.

### 2.4. Participants and Sample Size

To obtain data on the population of actively employed nurses at the secondary healthcare level in general and county hospitals in the Republic of Croatia, a list of healthcare institutions was retrieved from the official website of the Ministry of Health of the Republic of Croatia. The study was designed to include general and county hospitals across the Republic of Croatia in a geographically balanced manner. Geographical grouping of institutions was conducted according to the National Development Plan of the Ministry of Health of the Republic of Croatia, with hospitals divided into four geographical regions (Central and Northern, Eastern, Southern, and Western) to ensure proportional regional representation of the target population.

In order to define the total target population, all included institutions were contacted. Requests for information regarding the number of employed nurses at all educational levels were sent to hospital directors and nursing managers. Following this procedure, the total target population was defined as 7778 nurses employed across 22 hospitals. Nineteen of the 22 eligible hospitals participated in the study. These institutions represented approximately 93.9% of the target population and ensured coverage across all four geographical regions of Croatia.

After defining the target population (N = 7778), a representative sample of at least 367 respondents was planned, and proportional stratified sampling according to geographical region was applied. The sample size was calculated using the Cochran formula with a 95% confidence level and a maximum margin of error of ±5%. The calculated sample size for the finite population was 366.3 respondents, rounded to 367 participants.

After obtaining approval from the Ethics Committees of the participating institutions, meetings were conducted with nursing managers to organize the implementation of the study. Nurses employed in various clinical departments, including surgical departments, internal medicine departments, neurology, intensive care and anesthesiology units, psychiatry, pediatrics, and infectious disease departments, were invited to participate. Participation was voluntary and anonymous, and questionnaires were distributed to eligible nurses employed in the participating hospitals.

During the implementation of the study, a larger number of nurses than initially expected expressed willingness to participate, resulting in a final sample of 1139 respondents. Only fully completed questionnaires were included in the final analysis. This large sample size increased the precision of the estimates and reduced the margin of error to approximately 2.7%. Participants were recruited from multiple hospitals and various clinical departments, ensuring greater heterogeneity and representativeness of the sample.

### 2.5. Data Analysis

The results are presented descriptively using the mean, standard deviation, median, minimum, and maximum for continuous variables, while categorical variables are presented as absolute frequencies, relative frequencies (percentages), and cumulative percentages. The assumption of normal distribution for continuous variables was tested using the Shapiro–Wilk test. Given the results of the Shapiro–Wilk test (and regardless of the large sample size), non-parametric tests were used to test for differences between groups. This approach was chosen due to the nature of the outcome (knowledge test), for which the assumption of normality is often violated. Differences were analyzed with respect to education level, work department, frequency of working with patients with pressure ulcers, participation in pressure ulcer lectures, following guidelines, and searching for information about pressure ulcers. For post hoc analyses, the Dwass–Steel–Critchlow–Fligner (DSCF) test for multiple comparisons was used. The DSCF procedure is a conservative non-parametric method for pairwise comparisons that controls the family-wise Type I error rate across multiple tests [19]. The effect size was estimated using the epsilon-squared (ε^2^) statistic. The interpretation of the effect size was based on the following thresholds: ε^2^ ≈ 0.01 (small effect); ε^2^ ≈ 0.06 (medium effect); and ε^2^ ≥ 0.14 (large effect).

Participants were grouped within multiple hospitals. However, since the aim of the study was primarily to assess knowledge levels descriptively and to explore unadjusted differences between groups of respondents, the potential effect of grouping respondents within hospitals was not specifically modeled. This fact should be taken into account when interpreting the results. Categories with very small numbers of respondents are to be interpreted with caution, due to the limited statistical reliability of the estimates.

All collected data were transferred to Microsoft Office Excel, where they were coded for further processing. Statistical analyses were performed using IBM SPSS for Windows (Statistical Package for the Social Sciences), version 26.0 (IBM Corp.: Armonk, NY, USA), and R (version 4.3.1).

### 2.6. Research Ethics

The study was conducted in accordance with the ethical principles outlined in the Declaration of Helsinki (2013 revision). Participant anonymity and data confidentiality were ensured throughout the research process. Prior to participation, all respondents were informed about the purpose of the study, the intended use of the data, and the voluntary nature of their involvement, after which informed consent was obtained. Participants were also informed of their right to withdraw from the study at any time without any consequences. The study was carried out using a paper-based questionnaire. Ethical approval for the study was obtained from the relevant Institutional Ethics Committees prior to data collection. The list and reference numbers of the ethics committee approvals are provided in the Institutional Review Board Statement.

## 3. Results

### 3.1. Descriptive Statistics and Participant Characteristics

Table 1 presents the demographic characteristics of the study participants. The mean age was 37 years, and the mean work experience was 16.0 years. Both variables deviated significantly from a normal distribution (Shapiro–Wilk test, *p* < 0.001).

Among the 1139 respondents, women accounted for 85.0% of the sample. Most participants had completed secondary education (51.0%), followed by a bachelor’s degree (33.6%) and a master’s degree (15.2%), whereas only two participants held a doctoral degree. Respondents were primarily employed in internal medicine (27.4%) and surgical departments (23.4%), while the remaining participants were distributed across neurological, intensive care, and other clinical departments (Table 2).

The mean overall PZ-PUKT score was 64.1%. Knowledge was highest in the prevention domain (71.6%), whereas lower scores were observed for classification (60.5%) and wound appearance (56.4%). The wide score ranges observed across all domains indicate substantial variability in the participants’ knowledge levels (Table 3).

### 3.2. Differences in PZ-PUKT Results According to Level of Education

The descriptive results given in Table 4 indicate that knowledge increased with educational attainment. The overall PZ-PUKT score was higher among participants with a master’s degree (66.3%) and bachelor’s degree (65.6%) than among those with secondary education (62.5%). The highest scores were achieved in prevention knowledge across all educational groups, whereas lower scores were observed for wound classification and wound appearance.

The Kruskal–Wallis analysis revealed statistically significant differences between educational groups across all PZ-PUKT domains (Table 5). However, the effect sizes were uniformly very small. The weakest association was observed for prevention knowledge (χ^2^ = 9.13; *p* = 0.010; ε^2^ = 0.008), indicating a negligible practical effect despite statistical significance. Slightly larger—although still small—effect sizes were observed for wound classification, wound appearance, and overall PZ-PUKT scores (ε^2^ = 0.014–0.017).

Post hoc DSCF comparisons (Supplementary Table S1) indicated that these statistically significant differences were primarily attributable to lower scores among participants with secondary education. Bachelor’s degree holders achieved significantly higher scores in prevention knowledge (*p* = 0.026), while both bachelor’s and master’s degree holders scored higher in the pressure injury classification, wound description, and overall PZ-PUKT domains when compared with participants with secondary education (*p* < 0.001–0.009). No statistically significant differences were observed between the bachelor’s and master’s degree groups.

### 3.3. Differences in PZ-PUKT Results According to Working Department

For analytical purposes, individual work settings were grouped into seven departmental categories: Internal Medicine, Surgery, Neurology, Intensive Care Unit (ICU), Pediatrics, Emergency Department, and Palliative Care. The categories presented in Table 2 were aggregated into broader clinically relevant groups to facilitate comparisons of PI knowledge across work environments. For example, Intensive Care Units and Anesthesiology Departments were combined because they are commonly organised as a single clinical service within Croatian hospitals, although some respondents reported them separately. Departments with a small number of respondents were combined into clinically related categories or excluded from separate analyses.

This categorization enabled comparisons of PI knowledge across clinically relevant work environments.

Most respondents worked in the Internal Medicine (31.6%) and surgical (27.5%) departments, whereas the remaining participants were distributed across Neurology, ICU, Pediatrics, Emergency, and Palliative Care departments (Table 6).

The descriptive results suggested some variation in PZ-PUKT scores across departments (Table 7). Higher average scores were generally observed for those in the Pediatrics, ICU, and surgical departments, whereas lower scores were recorded in Internal Medicine and Emergency Department settings. Similar patterns were evident across prevention, classification, wound appearance, and overall knowledge scores.

The Kruskal–Wallis test results revealed statistically significant differences between departments across all analyzed PZ-PUKT domains (*p* < 0.001; Table 8). However, the effect sizes were small (ε^2^ ≈ 0.03–0.04), indicating that departmental differences accounted for only a limited proportion of the observed variability in knowledge scores. Therefore, although statistically significant, the practical magnitude of these differences should be interpreted with caution.

Post hoc DSCF analyses (Supplementary Table S2) indicated that significant pairwise differences were primarily related to comparisons with the Internal Medicine department, with the most consistent differences observed in prevention, classification, and overall knowledge scores when compared to Surgery, Pediatrics, and ICU settings. Significant differences in wound appearance knowledge were less frequent and mainly limited to comparisons between Surgery and Emergency Department staff. No other pairwise comparisons reached statistical significance.

### 3.4. Differences in PZ-PUKT Results According to Frequency of Working with PI

The descriptive results suggested a modest trend towards higher PZ-PUKT scores among participants who reported more frequent contact with patients with PI (Table 9). However, the differences between exposure groups were small, with overall mean scores ranging from 62.1% among respondents who never worked with patients with PI to 64.9% among those who encountered them several times per week. Similar patterns were observed across all knowledge domains.

The Kruskal–Wallis analysis did not identify a statistically significant difference according to frequency of working with patients with PI in any of the analyzed domains (χ^2^ = 3.98–9.00; df = 4; *p* = 0.061–0.409; Table 10). Although knowledge of wound classification showed the strongest trend (*p* = 0.061), the observed differences did not reach statistical significance.

### 3.5. Differences in PZ-PUKT Results According to Lecture Attendance

The descriptive results indicated higher PZ-PUKT scores among participants who had attended a PI lecture more recently, whereas the lowest scores were observed among respondents who had never attended such education (Table 11). The largest difference was observed for the overall PZ-PUKT score, which ranged from 48.0% in the “Never” group to 66.6% among participants who had attended a lecture within the previous year. Similar patterns were observed for classification and wound appearance knowledge.

The Kruskal–Wallis analysis identified statistically significant differences between lecture attendance groups across all PZ-PUKT domains (χ^2^ = 38.4–58.6; *p* < 0.001; Table 12). However, the effect sizes remained small (ε^2^ = 0.034–0.051), indicating that lecture attendance explained only a limited proportion of the variability in knowledge scores.

Post hoc DSCF analyses (Supplementary Table S3) demonstrated that significant pairwise differences were primarily attributable to lower scores among participants who had never attended a PI lecture. Respondents who reported attending a lecture within the previous year or the preceding 2–3 years generally achieved higher scores across all knowledge domains. Additional differences were observed between the most recently educated participants and those whose last lecture had occurred four or more years previously. No other pairwise comparisons reached statistical significance.

### 3.6. Differences in PZ-PUKT Results According to Reading the Professional Literature and Guidelines

The descriptive results indicated higher PZ-PUKT scores among participants who reported more recent engagement with the professional literature and guidelines (Table 13). The overall mean score ranged from 52.3% among respondents who had never consulted the professional literature to 66.7% among those who had done so within the previous year. Similar patterns were observed across all knowledge domains, particularly for classification and wound appearance.

The Kruskal–Wallis analysis identified statistically significant differences between groups across all PZ-PUKT domains (χ^2^ = 39.8–68.5; *p* < 0.001; Table 14). However, the effect sizes were small to modest (ε^2^ = 0.035–0.060), indicating that only a limited proportion of the variability in knowledge scores was associated with differences in literature-reading behavior.

Post hoc DSCF analyses (Supplementary Table S4) showed that significant differences were primarily observed between respondents who had consulted the professional literature within the previous year and those reporting longer intervals since their last reading or no previous engagement with the professional literature. Additional differences were identified in selected comparisons involving participants whose last exposure had occurred four or more years previously. No other pairwise comparisons reached statistical significance.

### 3.7. Differences in PZ-PUKT Results According to Literature Search on PI Within the Past Year

Participants who reported searching for information on PI within the previous year demonstrated higher PZ-PUKT scores across all knowledge domains than those who had not (Table 15). The largest difference was observed for classification knowledge (63.7% vs. 57.3%), while the overall score differed by 4.8 percentage points (66.6% vs. 61.8%).

The Mann–Whitney U analysis identified statistically significant differences between groups across all PZ-PUKT domains (*p* < 0.001; Table 16). However, the effect sizes were small (rpb = 0.133–0.214), indicating that information-seeking behavior was associated with only a limited proportion of the observed variability in knowledge scores. The largest effects were observed for classification knowledge and the overall PZ-PUKT score.

## 4. Discussion

Participants with secondary education generally achieved lower PZ-PUKT scores than those with bachelor’s or master’s degrees, consistent with previous studies reporting an association between higher educational attainment and greater knowledge of pressure injury prevention and management [15,20,21].

Nevertheless, the findings across countries remain inconsistent. While some studies have reported similar associations, others found no significant differences according to educational level or even higher scores among nurses with lower formal qualifications [22,23]. Such inconsistencies suggest that the relationship between education and knowledge may be influenced by contextual factors, including the structure of educational programs and opportunities for continuing professional development.

The present findings contribute to this body of evidence by demonstrating a similar pattern at the national level in Croatia.

However, although statistically significant, the observed effect sizes were very small—particularly for prevention knowledge—indicating the limited practical significance of the differences observed between educational groups.

Differences in knowledge scores were also observed across clinical departments. Higher scores were generally observed among nurses working in intensive care, surgical, and pediatric settings, whereas lower scores were recorded in emergency and internal medicine departments. One possible explanation for this observation is that differences in clinical exposure and organizational context may influence opportunities to acquire and maintain PI-related knowledge [24]. However, due to the cross-sectional study design, causal interpretations cannot be made. Although some differences between departments were observed, the median scores in most domains remained below the 70% threshold proposed by the original PZ-PUKT authors, except for those in the prevention domain. This pattern is broadly consistent with studies from several countries reporting moderate knowledge levels among nurses regarding the prevention and management of PI [25,26,27,28,29,30], although higher scores have occasionally been reported [31].

Importantly, the effect sizes for departmental differences were also small, indicating that work setting accounted for only a limited proportion of the variability in knowledge scores. This observation is consistent with previous research showing that departmental differences are neither universal nor consistently observed [22]. Furthermore, even individuals working in departments involving frequent exposure to pressure injuries may demonstrate insufficient knowledge levels when structured educational programs and standardized clinical protocols are lacking. No statistically significant association was found between the frequency of working with patients with pressure injuries and PZ-PUKT scores, although descriptive analyses suggested a slight upward trend in knowledge scores with increasing exposure. This finding is consistent with studies reporting weak or inconsistent relationships between clinical experience and PI knowledge [25,32]. One possible explanation is that clinical exposure alone may not be sufficient for knowledge acquisition without complementary educational activities and access to evidence-based resources [33]. The discrepancy between exposure and knowledge may reflect differences in the quality of clinical experience, opportunities for reflection, and engagement in continuing education [34]. Overall, the findings suggest that knowledge is influenced by multiple factors and cannot be explained by clinical exposure alone.

Participants who reported more recent attendance of PI educational activities achieved higher PZ-PUKT scores across all knowledge domains, when compared to those who had never attended such education. This finding is consistent with previous studies demonstrating an association between participation in educational activities and higher levels of PI knowledge [35]. Previous intervention studies have suggested that structured educational programs may improve knowledge and support evidence-based clinical practice [36,37,38]. However, as a cross-sectional study design was used, the observed associations cannot be interpreted as evidence of a causal effect of educational activities. Respondents who reported more recent attendance of educational activities achieved higher knowledge scores than those whose last such education had occurred several years earlier, consistent with previous studies suggesting that knowledge may decline over time without continued reinforcement and repeated exposure to educational content [34]. However, the observed effect sizes were small, indicating that participation in educational activities accounted for only a limited proportion of the variability in knowledge scores. This finding suggests that knowledge is influenced by multiple factors, including professional experience, clinical context, and engagement in continuing professional development [39]. Although participants who had never attended educational activities consistently achieved the lowest scores, the cross-sectional study design does not allow for conclusions regarding causality. It is also possible that individuals with greater professional interest or motivation are more likely to participate in educational activities and consequently demonstrate higher knowledge levels. Nevertheless, the observed associations are consistent with previous studies reporting higher knowledge scores among nurses who participate in educational programs [27,40]. The differences were most apparent in the classification and wound description domains, whereas prevention knowledge showed smaller variation between groups—a pattern that has also been described in previous research.

Respondents who reported engagement with the professional literature and clinical guidelines achieved higher PZ-PUKT scores than those who had not consulted such resources, with higher scores generally observed among participants who had accessed such information within the previous year.

This finding is consistent with studies emphasizing the importance of access to evidence-based resources for maintaining professional knowledge [13]. Previous intervention studies have shown that the implementation of evidence-based guidelines may improve preventive practices and reduce the incidence of PI [41,42,43,44]. However, such outcomes were not assessed in the present study; therefore, the clinical significance of the observed knowledge differences remains uncertain.

The association between recent literature consultation and higher scores may reflect knowledge maintenance through continued engagement with professional information [45]. As observed for educational activities, larger differences were found in the classification and wound description domains than in prevention knowledge. Previous studies have similarly suggested that more complex cognitive domains may require ongoing learning and updating of knowledge [20,46,47].

Participants who reported actively searching for information on pressure injuries during the previous year achieved higher knowledge scores across all PZ-PUKT domains. Nevertheless, the corresponding effect sizes were small, indicating that information-seeking behavior explains only a limited proportion of the variability in knowledge. This association is consistent with studies highlighting the roles of self-directed learning and information literacy in professional development [46,48,49].

Previous studies have suggested that access to up-to-date information and decision-support resources may facilitate evidence-based practice [31,38,50,51]. However, neither clinical behaviors nor patient outcomes were assessed in the present study, precluding any conclusions regarding the practical impacts of the observed knowledge differences.

The greater variability among respondents who did not seek information may reflect more heterogeneous knowledge levels within this group [52].

Importantly, the presented findings should not be interpreted as evidence that information-seeking behavior directly increases knowledge. It is equally plausible that nurses with greater knowledge, motivation, or professional interest are more likely to seek information. Future longitudinal studies are needed to clarify the direction and magnitude of these relationships. Nevertheless, the results support the value of fostering information literacy and facilitating access to evidence-based resources within clinical settings.

### Strengths, Limitations, and Future Directions

The main strengths of this study are its large national sample, multicenter design, and the use of the validated PZ-PUKT instrument. The inclusion of various clinical settings and factors related to knowledge enabled a comprehensive assessment of nurses’ knowledge regarding the prevention and management of PI at the national level.

This study has several limitations. Participation was voluntary, which may have introduced self-selection bias, as nurses with greater professional interest in PI prevention may have been more likely to participate. Due to the cross-sectional design and the absence of multivariable analyses, causal relationships and the independent contributions of individual factors to knowledge scores could not be determined. Potential clustering effects within hospitals were not analyzed. In addition, respondent fatigue associated with the 72-item PZ-PUKT questionnaire cannot be excluded.

Therefore, future studies should use multivariable, longitudinal, and intervention designs to identify independent determinants of PI knowledge, as well as to evaluate whether increased knowledge leads to improvements in clinical practice and patient outcomes.

## 5. Conclusions

This national study showed that nurses’ knowledge regarding pressure injury prevention and management varied according to educational level, work setting, participation in educational activities, engagement with professional literature, and active information-seeking behaviour. Higher knowledge scores were generally observed among nurses with higher educational attainment; however, important knowledge gaps remained evident, particularly in the domains of pressure injury classification and wound description. The findings highlight the importance of strengthening formal education and promoting continuous professional development related to pressure injury prevention and management. Given the cross-sectional design of the study, the observed differences should not be interpreted as causal relationships. Further longitudinal and interventional studies are needed to better understand the factors related to nurses’ knowledge and to evaluate the effectiveness of educational strategies in improving clinical practice and patient outcomes.

## Figures and Tables

**Table 1:** Descriptive and normality statistics for participant characteristics.

Variable	M	Median	SD	Min	Max	W	*p*
Age (years)	37.6	37	12.1	19	68	0.954	<0.001
Work experience (years)	16.0	14.0	12.1	0.0	49.0	0.928	<0.001

Note: M = mean; SD = standard deviation; W = Shapiro–Wilk W statistic; *p* = *p*-value.

**Table 2:** Sociodemographic, educational, and professional characteristics of participants.

Variable	Category	n (%)
Sex	Male	171 (15.0)
	Female	968 (85.0)
Education	Ph.D.	2 (0.2)
	Master’s degree	173 (15.2)
	Bachelor’s degree	383 (33.6)
	Secondary school	581 (51.0)
Department	Anesthesiology	9 (0.8)
	Gynecology	39 (3.4)
	Operating Room	28 (2.5)
	Internal	312 (27.4)
	ICU	81 (7.1)
	Surgery	267 (23.4)
	Neurology	139 (12.2)
	Pediatrics	65 (5.7)
	Psychiatry	30 (2.6)
	Other	169 (14.9)
Frequency of contact with patients with PI	Daily	500 (43.9)
	Several times per week	248 (21.8)
	Once per week	97 (8.5)
	Rarely	233 (20.5)
	Never	61 (5.4)
Last attended a lecture on PI	≤1 year	523 (45.9)
	>1 to <2 years	251 (22.0)
	2–3 years	200 (17.6)
	≥4 years	137 (12.0)
	Never	28 (2.5)
Last read professional literature on PI	≤1 year	599 (52.6)
	>1 to <2 years	252 (22.1)
	2–3 years	136 (11.9)
	≥4 years	124 (10.9)
	Never	28 (2.5)
Searched for information about PI on the web (last year)	Yes	559 (49.1)
	No	580 (50.9)

Note: For descriptive purposes, departments with a small number of respondents were grouped under the category “Other” to provide a more concise presentation of the sample.

**Table 3:** Descriptive and normality statistics for the knowledge results by domain.

Variable	M	Median	SD	Min	Max	W	*p*
PZ-PUKT: prevention	71.64	74.20	12.57	0.00	96.80	0.912	<0.001
PZ-PUKT: classification	60.47	61.90	16.20	0.00	100.00	0.983	<0.001
PZ-PUKT: wound description	56.37	60.00	14.98	0.00	90.00	0.971	<0.001
PZ-PUKT: total	64.14	65.30	12.37	0.00	91.70	0.958	<0.001

**Table 4:** Descriptive statistics for PZ-PUKT scores with regard to education level.

Variable	Education	N	M	Median	SD	Min	Max
PZ-PUKT: prevention	Ph.D.	2	79.0	79.0	2.26	77.4	80.6
	Master’s degree	173	73.2	74.2	11.84	25.8	96.8
	Bachelor’s degree	383	72.7	74.2	11.57	19.4	96.8
	Secondary school	581	70.5	71.0	13.32	0.0	93.5
PZ-PUKT: classification	Ph.D.	2	71.4	71.4	20.22	57.1	85.7
	Master’s degree	173	62.9	61.9	15.14	19.0	100.0
	Bachelor’s degree	383	62.3	61.9	14.96	14.3	100.0
	Secondary school	581	58.5	57.1	17.04	0.0	95.2
PZ-PUKT: wound description	Ph.D.	2	60.0	60.0	14.14	50.0	70.0
	Master’s degree	173	59.0	60.0	14.07	15.0	90.0
	Bachelor’s degree	383	58.2	60.0	13.95	10.0	90.0
	Secondary school	581	54.4	55.0	15.64	0.0	90.0
PZ-PUKT: total	Ph.D.	2	71.5	71.5	10.82	63.9	79.2
	Master’s degree	173	66.3	66.7	11.55	27.8	91.7
	Bachelor’s degree	383	65.6	66.7	11.06	27.8	91.7
	Secondary school	581	62.5	63.9	13.19	0.0	88.9

**Table 5:** Kruskal–Wallis statistics for PZ-PUKT scores by education level.

Variable	χ^2^	df	*p*	ε^2^
PZ-PUKT: prevention	9.13	2	0.010	0.008
PZ-PUKT: classification	15.89	2	<0.001	0.014
PZ-PUKT: wound description	19.23	2	<0.001	0.017
PZ-PUKT: total	19,47	2	<0.001	0.017

Note: χ^2^ = Kruskal–Wallis test statistic; df = degrees of freedom; *p* = *p*-value; ε^2^ = effect size (epsilon squared).

**Table 6:** Distribution of participants by aggregated departments.

Department (Aggregated Categories)	N	%
Internal	312	31.5
Surgery	272	27.5
Neurology	139	14.1
ICU	90	9.1
Pediatrics	65	6.6
Emergency Department	60	6.1
Palliative Care	50	5.1
Total	988 *	100.0

Note: * = For analytical purposes, individual departments were grouped into broader clinically relevant categories. Table 2 presents the original departmental distribution, whereas Table 6 presents the aggregated categories used in departmental comparisons.

**Table 7:** Descriptive statistics for PZ-PUKT scores by department.

Variable	Department	N	M	Med	SD	Min	Max
PZ-PUKT: prevention	Internal	312	70.1	71.0	11.80	0.0	93.5
	ICU	90	73.4	74.2	9.69	45.2	90.3
	Surgery	272	74.0	74.2	11.27	16.1	96.8
	Neurology	139	72.4	74.2	12.73	29.0	96.8
	Palliative Care	50	69.9	71.0	13.00	35.5	93.5
	Pediatrics	65	76.7	80.6	9.17	48.4	93.5
	Emergency Department	60	66.5	74.2	18.23	0.0	93.5
PZ-PUKT: classification	Internal	312	58.5	57.1	15.73	0.0	100.0
	ICU	90	65.5	66.7	14.04	28.6	90.5
	Surgery	272	61.8	61.9	16.00	0.0	100.0
	Neurology	139	62.7	61.9	15.58	4.8	90.5
	Palliative Care	50	56.1	57.1	13.17	14.3	81.0
	Pediatrics	65	64.3	66.7	15.21	19.0	95.2
	Emergency Department	60	58.9	61.9	20.43	0.0	95.2
PZ-PUKT: wound description	Internal	312	54.1	55.0	14.59	10	90
	ICU	90	59.1	60.0	14.28	25	85
	Surgery	272	59.4	60.0	13.47	10	90
	Neurology	139	57.2	60.0	15.77	0	90
	Palliative Care	50	58.0	60.0	14,36	10	85
	Pediatrics	65	57.5	60,0	16.40	10	85
	Emergency Department	60	52.2	55.0	15.63	0	80
PZ-PUKT: total	Internal	312	62.3	63.9	11.77	2.8	88.9
	ICU	90	67.1	68.1	10.29	38.9	86.1
	Surgery	272	66.4	66.7	11.25	9.7	91.7
	Neurology	139	65.4	66.7	12.66	20.8	91.7
	Palliative Care	50	62.6	63.9	11.58	25.0	80.6
	Pediatrics	65	67.8	68.1	11.52	29.2	84.7
	Emergency Department	60	60.3	61.8	16.55	0.0	87.5

**Table 8:** Kruskal–Wallis statistics for PZ-PUKT scores between groups.

Variable	χ^2^	df	*p*	ε^2^
PZ-PUKT: prevention	38.6	6	<0.001	0.039
PZ-PUKT: classification	29.7	6	<0.001	0.030
PZ-PUKT: wound description	29.6	6	<0.001	0.030
PZ-PUKT: total	37.5	6	<0.001	0.038

Note: χ^2^ = Kruskal–Wallis test statistic; df = degrees of freedom; *p* = *p*-value; ε^2^ = effect size (epsilon squared).

**Table 9:** Descriptive statistics for PZ-PUKT scores by frequency of working with patients with PI.

Variable	Frequency	N	M	Med	SD	Min	Max
PZ-PUKT: prevention	Never	61	70.1	71.0	14.73	25.8	93.5
	Rarely	233	71.3	74.2	13.33	0.0	96.8
	Once a week	97	71.0	71.0	9.18	41.9	90.3
	Several times a week	248	72.9	74.2	10.93	19.4	93.5
	Daily	500	71.5	74.2	13.24	0.0	96.8
PZ-PUKT: classification	Never	61	57.7	57.1	17.22	9.5	95.2
	Rarely	233	58.1	57.1	16.59	0.0	100.0
	Once a week	97	61.2	61.9	13.10	28.6	95.2
	Several times a week	248	61.7	61.9	16.73	14.3	100.0
	Daily	500	61.2	61.9	16.07	0.0	100.0
PZ-PUKT: wound description	Never	61	54.5	55.0	17.50	5	90
	Rarely	233	56.1	60.0	15.74	10	90
	Once a week	97	54.9	55.0	13.75	10	85
	Several times a week	248	56.1	55.0	14.06	10	90
	Daily	500	57.1	60.0	14.96	0	90
PZ-PUKT: total	Never	61	62.1	63.9	14.40	23.6	87.5
	Rarely	233	63.2	65.3	12.99	2.8	91.7
	Once a week	97	63.7	63.9	9.30	34.7	86.1
	Several times a week	248	64.9	65.3	11.54	27.8	88.9
	Daily	500	64.5	65.3	12.71	0.0	91.7

**Table 10:** Kruskal–Wallis statistics for PZ-PUKT scores by frequency of working with patients with PI.

Variable	χ^2^	df	*p*	ε^2^
PZ-PUKT: prevention	3.98	4	0.409	0.0035
PZ-PUKT: classification	9.00	4	0.061	00079
PZ-PUKT: wound description	4.61	4	0.329	0.0041
PZ-PUKT: total	5.65	4	0.227	0.0050

Note: χ^2^ = Kruskal–Wallis test statistic; df = degrees of freedom; *p* = *p*-value; ε^2^ = effect size (epsilon squared).

**Table 11:** Descriptive statistics for PZ-PUKT scores by time since attending a lecture on PI.

Variable	Last Lecture	N	M	Median	SD	Min	Max
PZ-PUKT: prevention	≤1 year	523	73.4	74.2	11.4	29.0	96.8
	>1 to <2 years	251	71.4	74.2	10.7	32.3	93.5
	2–3 years	200	72.4	74.2	10.6	25.8	96.8
	≥4 years	137	67.2	71.0	15.7	0.0	93.5
	Never	28	56.1	62.9	24.0	0.0	90.3
PZ-PUKT: classification	≤1 year	523	63.7	61.9	15.7	4.8	100.0
	>1 to <2 years	251	60.3	61.9	14.5	19.0	100.0
	2–3 years	200	58.8	57.1	14.9	19.0	95.2
	≥4 years	137	54.3	57.1	17.1	0.0	100.0
	Never	28	43.2	42.9	23.0	0.0	85.7
PZ-PUKT: wound description	≤1 year	523	59.0	60.0	13.7	10	90
	>1 to <2 years	251	56.2	60.0	14.1	0	85
	2–3 years	200	54.5	55.0	14.2	15	90
	≥4 years	137	52.5	55.0	18.2	0	85
	Never	28	40.4	40.0	18.4	0	75
PZ-PUKT: total	≤1 year	523	66.6	66.7	11.4	20.8	91.7
	>1 to <2 years	251	63.9	65.3	10.8	22.2	88.9
	2–3 years	200	63.5	64.6	10.5	36.1	91.7
	≥4 years	137	59.4	62.5	14.9	0.0	88.9
	Never	28	48.0	52.1	20.8	0.0	81.9

**Table 12:** Kruskal–Wallis statistics for PZ-PUKT scores between groups by time since attending a lecture on PI.

Variable	χ^2^	df	*p*	ε^2^
PZ-PUKT: prevention	38.4	4	<0.001	0.0338
PZ-PUKT: classification	58.2	4	<0.001	0.0511
PZ-PUKT: wound description	44.5	4	<0.001	0.0391
PZ-PUKT: total	58.6	4	<0.001	0.0515

Note: χ^2^ = Kruskal–Wallis test statistic; df = degrees of freedom; *p* = *p*-value; ε^2^ = effect size (epsilon squared).

**Table 13:** Descriptive statistics for PZ-PUKT scores by time since reading the professional literature on PI.

Variable	Last Reading	N	M	Med	SD	Min	Max
PZ-PUKT: prevention	≤1 year	599	73.6	74.2	10.9	29.0	96.8
	>1 to <2 years	252	71.7	74.2	11.2	25.8	96.8
	2–3 years	136	70.2	71.0	12.2	19.4	93.5
	≥4 years	124	65.9	71.0	16.4	0.0	93.5
	Never	28	61.6	64.5	23.4	00	93.5
PZ-PUKT: classification	≤1 year	599	63.6	61.9	15.2	95	100.0
	>1 to <2 years	252	60.0	61.9	14.9	4.8	100.0
	2–3 years	136	57.2	57.1	15.0	23.8	90.5
	≥4 years	124	53.2	57.1	17.5	0.0	95.2
	Never	28	45.9	47.6	24.6	0.0	100.0
PZ-PUKT: wound description	≤1 year	599	59.3	60.0	13.0	0	90
	>1 to <2 years	252	55.5	55.0	14.9	10	90
	2–3 years	136	53.5	55.0	15.1	10	85
	≥4 years	124	49.8	55.0	17.9	0	80
	Never	28	44.5	47.5	21.2	0	75
PZ-PUKT: total	≤1 year	599	66.7	66.7	10.8	22.2	91,7
	>1 to <2 years	252	63.8	65.3	11.2	20.8	91.7
	2–3 years	136	61.7	61.8	11.8	27.8	86.1
	≥4 years	124	57.7	62.5	15.1	0.0	84.7
	Never	28	52.3	54.2	21.7	0.0	88.9

**Table 14:** Kruskal–Wallis statistics for PZ-PUKT scores by time since reading guidelines and the professional literature on PI.

Variable	χ^2^	df	*p*	ε^2^
PZ-PUKT: prevention	39.8	4	<0.001	0.0349
PZ-PUKT: classification	60.5	4	<0.001	0.0532
PZ-PUKT: wound description	53.2	4	<0.001	0.0467
PZ-PUKT: total	68.5	4	<0.001	0.0602

Note: χ^2^ = Kruskal–Wallis test statistic; df = degrees of freedom; *p* = *p*-value; ε^2^ = effect size (epsilon squared).

**Table 15:** Descriptive statistics for PZ-PUKT scores by searching for information on PI within the past year.

Variable	Literature Search	N	M	Median	SD	Min	Max
PZ-PUKT: prevention	Yes	559	73.6	74.2	10.4	29.0	96.8
	No	580	69.8	71.0	14.1	0.0	93.5
PZ-PUKT: classification	Yes	559	63.7	61.9	15.3	14.3	100.0
	No	580	57.3	57.1	16.4	0.0	100.0
PZ-PUKT: wound description	Yes	559	58.6	60.0	13.7	10	90
	No	580	54.2	55.0	15.9	0	90
PZ-PUKT: total	Yes	559	66.6	66.7	10.8	31.9	91.7
	No	580	61.8	63.9	13.3	0.0	88.9

**Table 16:** Mann–Whitney U test statistics for PZ-PUKT scores by searching for information on PI within the past year.

Variable	U	*p*	Mean Difference	r_rb_
PZ-PUKT: prevention	140,606	<0.001	3.20	−0.133
PZ-PUKT: classification	127,453	<0.001	4.80	−0.214
PZ-PUKT: wound description	137,335	<0.001	5.00	−0.153
PZ-PUKT: total	130,068	<0.001	4.10	−0.198

Note: U = Mann–Whitney U statistic; r_rb_ = rank-biserial correlation (effect size). A positive mean difference indicates higher values in the “Yes” group.

## Data Availability

The data presented in this study are available from the corresponding author upon reasonable request, due to the need to protect the anonymity of participants and the institutions involved in the study. Additional materials supporting the findings are provided in the Supplementary Materials accompanying this article.

## Supplementary Materials

The following supporting information can be downloaded at https://www.mdpi.com/article/10.3390/healthcare14131948/s1. Table S1. Statistically significant Dwass–Steel–Critchlow–Fligner (DSCF) post hoc comparisons of PZ–PUKT scores by education level; Table S2. Statistically significant DSCF post hoc comparisons of PZ–PUKT scores by department; Table S3. Statistically significant DSCF post hoc comparisons of PZ–PUKT scores by time since the last lecture; Table S4. Statistically significant DSCF post hoc comparisons of PZ–PUKT scores by time since last reading of professional literature.

## Abbreviations

The following abbreviations are used in this manuscript:

PZ-PUKT	Pieper–Zulkowski Pressure Ulcer Knowledge Test
PI	Pressure injury
KR-20	Kuder–Richardson formula 20
ICU	Intensive Care Unit

## References

[1] Xie Z.-Q., Tao X.-M., Wang Z.-Q., Du Y.-Y., Yi L.-X., Xie C., Yi H.-X., Zhang M., Xiong W.-Y., Chen S.-H. (2025). Pressure Injury Incidence and Quality of Care Index (1990–2021): An Analysis of Trends and Health Inequalities Based on the Study of Global Burden of Disease 2021. Adv. Wound Care.

[2] Kaya F.O., Şimşek S. (2025). Clinical and Systemic Factors Associated with Pressure Ulcer Development: A Retrospective Evaluation of 339 Patients in a Palliative Care Unit. Front. Med..

[3] Krupová L., Pokorná A., Krupa M., Benešová K. (2025). Comprehensive Cost-of-illness Analysis of Pressure Ulcer Treatment: A Real-world Study at a Czech University Hospital. Int. Wound J..

[4] Al-Qudimat A.R., Maabreh A.H., Shtayat H., Khaleel M.A., Allatayfeh J.M., Iblasi A.S. (2024). Prevention of Pressure Injuries and Nursing Interventions in Critical Care Settings: A Synthesis Without Meta-Analysis (SWiM). Chronic Wound Care Manag. Res..

[5] McMahon J., McInnes E., Wan C.S., Straiton N., Lam L., Rodgers J., Dickson J., Fulbrook P. (2026). Effectiveness of Organisational Strategies for Pressure Injury Prevention and Treatment in Acute Hospital Settings: A Systematic Review. J. Adv. Nurs..

[6] Domitrović D.L., Relić D., Britvić A. (2018). Nursing Education in Croatia. Liječnički Vjesn..

[7] Kurtovic B., Friganovic A., Cukljek S., Vidmanic S., Stievano A. (2021). The Development of the Nursing Profession and Nursing Education in Croatia. J. Prof. Nurs..

[8] Lewis L.S., Rebeschi L.M., Hunt E. (2022). Nursing Education Practice Update 2022: Competency-Based Education in Nursing. SAGE Open Nurs..

[9] Hill J., Gratton N., Kulkarni A., Hamer O., Harrison J., Harris C., Chesters J., Duddy E., Collins L., Clegg A. (2024). The Effectiveness of Evidence-based Healthcare Educational Interventions on Healthcare Professionals’ Knowledge, Skills, Attitudes, Professional Practice and Healthcare Outcomes: Systematic Review and Meta-analysis. J. Eval. Clin. Pract..

[10] Ramis M.-A., Chang A., Conway A., Lim D., Munday J., Nissen L. (2019). Theory-Based Strategies for Teaching Evidence-Based Practice to Undergraduate Health Students: A Systematic Review. BMC Med. Educ..

[11] Miller G.E. (1990). The Assessment of Clinical Skills/Competence/Performance. Acad. Med..

[12] Kitamura J.C., Nicolosi J.T., Paggiaro A.O., Fernandes De Carvalho V. (2023). Educational Interventions on Preventing Pressure Injuries Targeted at Nurses: Systematic Review and Meta-Analysis. Br. J. Nurs..

[13] Wan C.S., Cheng H., Musgrave-Takeda M., Liu M.G., Tobiano G., McMahon J., McInnes E. (2023). Barriers and Facilitators to Implementing Pressure Injury Prevention and Management Guidelines in Acute Care: A Mixed-Methods Systematic Review. Int. J. Nurs. Stud..

[14] Araujo S.M., Sousa P., Dutra I. (2020). Clinical Decision Support Systems for Pressure Ulcer Management: Systematic Review. JMIR Med. Inform..

[15] Alshahrani B., Middleton R., Rolls K., Sim J. (2023). Critical Care Nurses’ Knowledge and Attitudes toward Pressure Injury Prevention: A Pre and Post Intervention Study. Intensive Crit. Care Nurs..

[16] Šepl Plentaj A., Žulec M. (2022). Nurses’ Knowledge About Wound Care: Croatian Perspective. Croat. Nurs. J. (Online).

[17] Žepina Puzić A., Filej B., Žulec M., Bušac V., Bertić Ž., Jurčev Savičević A. (2025). Initial Validation and Psychometric Properties of the Croatian Version of the Pieper–Zulkowski Pressure Ulcer Knowledge Test. Healthcare.

[18] Fulbrook P., Lawrence P., Miles S. (2019). Australian Nurses’ Knowledge of Pressure Injury Prevention and Management: A Cross-Sectional Survey. J. Wound Ostomy Cont. Nurs..

[19] Hsu J. (1996). Multiple Comparisons: Theory and Methods.

[20] Žepina Puzić A., Žulec M., Bušac V., Bertić Ž., Filej B. (2025). Impact of Formal and Lifelong Education on Croatian Nurses’ Knowledge of Pressure Ulcers: A Cross-Sectional Study. Nurs. Rep..

[21] Nieto-García A., Nieto-García L., Roca-Biosca M.A., Moreiro-Barroso M.T., Carpio-Pérez A., Alonso-Sardón M. (2026). Pressure Injury Prevention Knowledge in Nursing Professionals. J. Tissue Viability.

[22] Sayar S., Aşkın Ceran M., Demir A. (2022). Determining the Pressure Injury and Staging Knowledge of Nurses at a Hospital in Turkey. J. Tissue Viability.

[23] Grešš Halász B., Bérešová A., Tkáčová Ľ., Magurová D., Lizáková Ľ. (2021). Nurses’ Knowledge and Attitudes towards Prevention of Pressure Ulcers. Int. J. Environ. Res. Public Health.

[24] Li Z., Lin F., Thalib L., Chaboyer W. (2020). Global Prevalence and Incidence of Pressure Injuries in Hospitalised Adult Patients: A Systematic Review and Meta-Analysis. Int. J. Nurs. Stud..

[25] Klaas N., Serebro R.-L. (2024). Intensive Care Nurses’ Knowledge of Pressure Injury Prevention. BMC Nurs..

[26] Kısacık Ö.G., Sönmez M. (2020). Pressure Ulcers Prevention: Turkish Nursing Students’ Knowledge and Attitudes and Influencing Factors. J. Tissue Viability.

[27] Tirgari B., Mirshekari L., Forouzi M.A. (2018). Pressure Injury Prevention: Knowledge and Attitudes of Iranian Intensive Care Nurses. Adv. Skin. Wound Care.

[28] Coventry L., Towell-Barnard A., Winderbaum J., Walsh N., Jenkins M., Beeckman D. (2024). Nurse Knowledge, Attitudes, and Barriers to Pressure Injuries: A Cross-Sectional Study in an Australian Metropolitan Teaching Hospital. J. Tissue Viability.

[29] Batiha A.-M. (2018). Critical Care Nurses Knowledge, Attitudes, and Perceived Barriers towards Pressure Injuries Prevention. Int. J. Adv. Nurs. Sci..

[30] John A.M., Nayak K.R.U., Lobo G., Thaleppaddy M. (2024). Assessment of Pressure Injury Knowledge Using PZ-PUKT Questionnaire among Indian Nurses and the Evaluation of Impact of an Educational Intervention on the Knowledge Scores: A Quasi-Experimental Study. J. Tissue Viability.

[31] Chao W., Hsu M., Chen S., Wu T., Kuo Y., Huang Z., Wu Y. (2024). Traditional Chinese-version Reliability Test of the Pieper-Zulkowski Pressure Ulcer Knowledge: Psychometric and Assessment. Int. Wound J..

[32] Wu J., Wang B., Zhu L., Jia X. (2022). Nurses’ Knowledge on Pressure Ulcer Prevention: An Updated Systematic Review and Meta-Analysis Based on the Pressure Ulcer Knowledge Assessment Tool. Front. Public Health.

[33] Tayyib N. (2025). Assessment of Registered Nurses’ Knowledge of Pressure Injury Prevention and the Impact of Training Recency: A Cross-Sectional Study. Nurs. Rep..

[34] Inayat F., Javed H.S., Inayat S., Khan N., Shakir J., Khalid A., Ullah M.J. (2025). Knowledge, Attitude, and Practice among Nurses Regarding the Prevention of Pressure Ulcers in a Tertiary Care Hospital: A Cross-Sectional Study. Sci. Rep..

[35] Khojastehfar S., Najafi Ghezeljeh T., Haghani S. (2020). Factors Related to Knowledge, Attitude, and Practice of Nurses in Intensive Care Unit in the Area of Pressure Ulcer Prevention: A Multicenter Study. J. Tissue Viability.

[36] Karimian M., Khalighi E., Salimi E., Borji M., Tarjoman A., Mahmoudi Y. (2020). The Effect of Educational Intervention on the Knowledge and Attitude of Intensive Care Nurses in the Prevention of Pressure Ulcers. Int. J. Risk Saf. Med..

[37] Saibertová S., Búřilová P., Krupová L., Dolanová D., Riad A., Pokorná A. (2025). Nurses’ Knowledge of Pressure Ulcer Management Related to Monitoring the Financial Costs of Pressure Ulcer Treatment: A Prospective Intervention Study. J. Wound Manag..

[38] Gaspar S., Peralta M., Marques A., Budri A., Gaspar De Matos M. (2019). Effectiveness on Hospital-acquired Pressure Ulcers Prevention: A Systematic Review. Int. Wound J..

[39] Latimer S., Chaboyer W., Gillespie B. (2016). Pressure Injury Prevention Strategies in Acute Medical Inpatients: An Observational Study. Contemp. Nurse.

[40] Gul A., Andsoy I.I., Ozkaya B., Zeydan A. (2017). A Descriptive, Cross-Sectional Survey of Turkish Nurses’ Knowledge of Pressure Ulcer Risk, Prevention, and Staging. Ostomy Wound Manag..

[41] Jafary M., Adibi H., Shayanfard K., Zohdi M., Godarzi Z., Yaseri M., Najafpour Z. (2018). Pressure Ulcer Rate in Multidisciplinary Hospital Units After Multifactorial Intervention: A Stepped-Wedge, Cluster Randomized Controlled Trial. J. Patient Saf..

[42] Curtis Á., Derwin R., Milne G., Connor A.M., Nugent L., Moore Z. (2025). What Is the Impact of Care Bundles on the Prevalence or Incidence of Pressure Ulcers Among At-Risk Adults in the Acute Care Setting? A Systematic Review. Int. Wound J..

[43] Avşar P., Karadağ A. (2018). Efficacy and Cost-Effectiveness Analysis of Evidence-Based Nursing Interventions to Maintain Tissue Integrity to Prevent Pressure Ulcers and Incontinence-Associated Dermatitis. Worldviews Evid. Based Nurs..

[44] Moore Z., Cowman S. (2012). Pressure Ulcer Prevalence and Prevention Practices in Care of the Older Person in the Republic of Ireland. J. Clin. Nurs..

[45] Ding Y., Qian J., Zhou Y., Zhang Y. (2024). Effect of E-LEARNING Program for Improving Nurse Knowledge and Practice towards Managing Pressure Injuries: A Systematic Review and Meta-analysis. Nurs. Open.

[46] Ebi W.E., Hirko G.F., Mijena D.A. (2019). Nurses’ Knowledge to Pressure Ulcer Prevention in Public Hospitals in Wollega: A Cross-Sectional Study Design. BMC Nurs..

[47] Aiman U., Saddique H., Jabeen R. (2024). Nursing knowledge and practice regarding the prevention of pressure ulcer. Biol. Clin. Sci. Res. J..

[48] Bektas İ., Kudubes A.A., Ayar D., Bektas M. (2025). The Level of Nursing Students’ Self-Regulated Learning and Academic Locus of Control Predicting Self-Confidence and Anxiety in Clinical Decision-Making. Nurse Educ. Today.

[49] Rababah J.A., Al-Hammouri M.M. (2024). Predictors of Jordanian Registered Nurses’ Clinical Decision-Making: The Role of Self-Directed Learning and Personal Characteristics. Nurs. Forum.

[50] Huang H.-Y., Lee T.-T., Hsu T.-C., Mills M.E., Tzeng I.-S. (2020). Evaluation of the Pressure Injury Prevention Information System. CIN Comput. Inform. Nurs..

[51] Cordina J., Rolls K., Sim J. (2025). Nurses’ Clinical Decision-Making About Pressure Injury Prevention in Hospital Settings: A Scoping Review. J. Adv. Nurs..

[52] Field A. (2009). Discovering Statistics Using SPSS: And Sex, Drugs and Rock “n” Roll.

