# Supplementary material for: Assessment of Nurses’ Knowledge Regarding Pressure Injury: A National Multicenter Cross-Sectional Study
Source: Healthcare (Basel). 2026 Jul 1;14(13):1948. doi: 10.3390/healthcare14131948 (PMC13361861; doi:10.3390/healthcare14131948)
Supplement: Supplementary file 1 [file healthcare-14-01948-s001.zip › healthcare-4315274-supplementary.pdf]

Supplementary Tables

Table S1. Statistically significant Dwass-Steel-Critchlow-Fligner (DSCF) post hoc comparisons of PZ–PUKT scores by education level.

Variable	Group comparison	W	p
PZ-PUKT: prevention	Bachelor's – Secondary education	−3.670	0.026
PZ-PUKT: classification	Master's – Secondary education	−4.153	0.009
	Bachelor's – Secondary education	−4.887	0.002
PZ-PUKT: wound description	Master's – Secondary education	−5.229	<0.001
	Bachelor's – Secondary education	−4.724	0.002
PZ-PUKT: total	Master's – Secondary education	−4.951	0.001
	Bachelor's – Secondary education	−5.104	<0.001

Note: W = DSCF test statistic; p = p-value

Table S2. Statistically significant DSCF post hoc comparisons of PZ–PUKT scores by department.

Variable	Department comparison	W	p
PZ-PUKT: prevention	Internal Medicine – Surgery	6.288	< 0.001
	Internal Medicine – Paediatrics	6.707	< 0.001
	Palliative care – Paediatrics	4.293	0.039
	Paediatrics – Emergency Department	−5.148	0.005
PZ-PUKT: classification	Internal Medicine – Intensive Care Unit	5.713	0.001
	Internal Medicine – Neurology	4.181	0.049
	Internal Medicine – Paediatrics	4.574	0.021

Variable	Department comparison	W	p
	Intensive Care Unit – Palliative care	−5.243	0.004
	Palliative care – Paediatrics	4.677	0.016
PZ-PUKT: wound description	Internal Medicine – Surgery	6.705	< 0.001
	Surgery – Emergency Department	−4.711	0.015
PZ-PUKT: total	Internal Medicine – Intensive Care Unit	5.393	0.003
	Internal Medicine – Surgery	6.478	< 0.001
	Internal Medicine – Neurology	4.299	0.038
	Internal Medicine – Paediatrics	5.300	0.003

Note: W = DSCF test statistic; p = p-value. Only statistically significant comparisons are shown (p < 0.05)

Table S3. Statistically significant DSCF post hoc comparisons of PZ–PUKT scores by time since the last lecture.

Varijable	Group comparison	W	p
PZ-PUKT: prevention	2–3 years – ≥ 4 years	−4.06	0.033
	2–3 years – Never	−5.66	< 0.001
	≥ 4 years – 1 year or less	6.14	< 0.001
	1 year or less – Never	−6.19	< 0.001
	Never – > 1 and < 2 years	5.22	0.002
PZ-PUKT: classification	2–3 years – 1 year or less	5.13	0.003
	2–3 years – Never	−4.95	0.004
	≥ 4 years – 1 year or less	8.21	< 0.001
	≥ 4 years – > 1 and < 2 years	4.66	0.009
	1 year or less – Never	−6.79	< 0.001
	1 year or less – > 1 and < 2 years	−4.33	0.019
	Never – > 1 and < 2 years	5.54	< 0.001

Varijable	Group comparison	W	p
PZ-PUKT: wound description	2–3 years – 1 year or less	5.50	< 0.001
	2–3 years – Never	−5.52	< 0.001
	≥ 4 years – 1 year or less	4.80	0.006
	≥ 4 years – Never	−4.40	0.016
	1 year or less – Never	−7.39	< 0.001
	Never – > 1 and < 2 years	6.30	< 0.001
PZ-PUKT: total	2–3 years – 1 year or less	4.98	0.004
	2–3 years – Never	−6.11	< 0.001
	≥ 4 years – 1 year or less	7.45	< 0.001
	≥ 4 years – Never	−4.18	0.026
	≥ 4 years – > 1 and < 2 years	4.14	0.028
	1 year or less – Never	−7.51	< 0.001
	1 year or less – > 1 and < 2 years	−4.40	0.016
	Never – > 1 and < 2 years	6.33	< 0.001

Note: W = DSCF test statistic; p = p-value

Table S4. Statistically significant DSCF post hoc comparisons of PZ–PUKT scores by time since last reading of professional literature.

Varijable	Group comparison	W	p
PZ-PUKT: prevention	2–3 years – 1 year or less	4.40	0,016
	≥ 4 years – 1 year or less	7.31	< 0.001
	≥ 4 years – > 1 and < 2 years	4.85	0.005
	1 year or less – Never	−4.57	0.011
PZ-PUKT: classification	2–3 years – 1 year or less	6.34	< 0.001
	≥ 4 years – 1 year or less	8.16	< 0.001
	≥ 4 years – > 1 and < 2 years	4.52	0.012

Varijable	Group comparison	W	p
	1 year or less – Never	−5.98	< 0.001
	1 year or less – > 1 and < 2 years	−4.53	0.012
	Never – > 1 and < 2 years	4.73	0.007
PZ-PUKT: wound description	2–3 years – 1 year or less	6.23	< 0.001
	≥ 4 years – 1 year or less	7.44	< 0.001
	1 year or less – Never	−5.33	0.002
	1 year or less – > 1 and < 2 years	−5.28	0.002
PZ-PUKT: total	2–3 years – 1 year or less	6.75	< 0.001
	≥ 4 years – 1 year or less	8.94	< 0.001
	≥ 4 years – > 1 and < 2 years	5.07	0.003
	1 year or less – Never	−5.83	< 0.001
	1 year or less – > 1 and < 2 years	−5.07	0.003
	Never – > 1 and < 2 years	4.48	0.013

Note: W = DSCF test statistic; p = p-value
